# Boosting Output Performance of Triboelectric Nanogenerator via Mutual Coupling Effects Enabled Photon‐Carriers and Plasmon

**DOI:** 10.1002/advs.202103957

**Published:** 2021-11-23

**Authors:** Xin Chen, Yanjun Zhao, Fayang Wang, Daqiao Tong, Lingxiao Gao, Dongxiao Li, Liangke Wu, Xiaojing Mu, Ya Yang

**Affiliations:** ^1^ Key Laboratory of Optoelectronic Technology & Systems Ministry of Education International R & D center of Micro‐nano Systems and New Materials Technology Chongqing University Chongqing 400044 China; ^2^ CAS Center for Excellence in Nanoscience Beijing Key Laboratory of Micro‐nano Energy and Sensor Beijing Institute of Nanoenergy and Nanosystems Chinese Academy of Sciences Beijing 101400 China; ^3^ School of Mechanical Engineering Hebei University of Technology Tianjin 300401 P. R. China; ^4^ College of Aerospace Engineering Chongqing University Chongqing 400044 China

**Keywords:** mutual coupling effects, output performance, photon‐generated carriers, surface plasmon resonance, triboelectric nanogenerators

## Abstract

Boosting the output performance of triboelectric nanogenerators via some unique methods is always a meaningful way to speed up their commercialization. However, the available approach to boost performance is mainly restricted to one physics effect based and the basic research of boosting performance via mutual coupling effects is little research. Herein, a new strategy is creatively proposed based on charge traps from mutual coupling effects, generated from g‐C_3_N_4_/MXene‐Au composites, to further promote the output performance of triboelectric nanogenerator. It is found that photon‐generated carriers coupling surface plasmon effect enables composites filled into tribo‐material with visible light is an excellent value in boosting performance. The charge traps from mutual coupling effects for boosting performance are analyzed theoretically and verified by experiments. The output power of boosting‐triboelectric nanogenerator (TENG) achieves a sixfold enhancement (20 mW) of normal TENG with polydimethylsiloxane (PDMS) in ambient conditions. This work provides a profound understanding of the working mechanism of mutual coupling effects boosting the performance of TENG and an effective way for promoting TENG output.

## Graphical abstract

A novel strategy based on charge traps from mutual coupling effects enabled photon‐carriers and plasmon generated from g‐C_3_N_4_/MXene‐Au composites has been utilized to further promote the output performance of triboelectric nanogenerator.

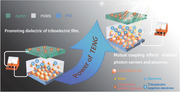

## 1 Introduction

Sustainable energy supply has always been one of the concerned topics in the perpetual historical flow. The triboelectric nanogenerator (TENG) which was put forward by Wang and co‐workers in 2012, has been widely verified as the credible power source for the sensor nodes and self‐powered sensors. The output electricity is one of the critical factors for TENG applied in smart Internet, a lot of hard work has been done to achieve the goal of output power reaching mW,^[^ ^1^, ^2^, ^3^, ^4^, ^5^, ^6^, ^7^, ^8^ ^]^ which is enough for autonomous sensor nodes. Thus, a variety of methods including surface functionalization, nanoparticles dopant, and chemical modification have been proposed.^[^ ^9^, ^10^, ^11^, ^12^, ^13^, ^14^ ^]^ However, these methods mainly changed surface properties of the triboelectric films and lost its potency after a while. In addition, most reported studies merely adopt one mechanism to enhance the output electric, limiting the power density promotion of the TENG.^[^ ^15^, ^16^, ^17^, ^18^, ^19^, ^20^, ^21^ ^]^ Thus, there is an urgent need for a new method to break the limitations of traditional methods.

The photon‐generated carriers have been widely used in the energy field and opened up a wide range of applications in the memory devices, the solar cells, especially in the research area of photocatalytic. There is a plausible consequence that the semiconductor materials and the 2D nanomaterials can generate carriers through light irradiation.^[^ ^22^, ^23^, ^24^, ^25^, ^26^, ^27^ ^]^ Some researchers have adopted the semiconductor materials such as TiO_2_ as the triboelectric layer. These studies verify that the light can regulate the output electricity of TENG as the materials are sensitive to the light, but photon‐generated carriers are not involved in the process of regulating output electricity of the TENG, and the TENG utilized these materials possess low power. And our research group has studied the hot electron–hole pairs from plasmon decay as charge traps can enhance the output electricity of TENG, while the regulation excitation is restricted to the illumination bandwidth.^[^ ^28^ ^]^ Thus, a new mechanism with a more vital boost ability is demanded.

**Figure 1:**
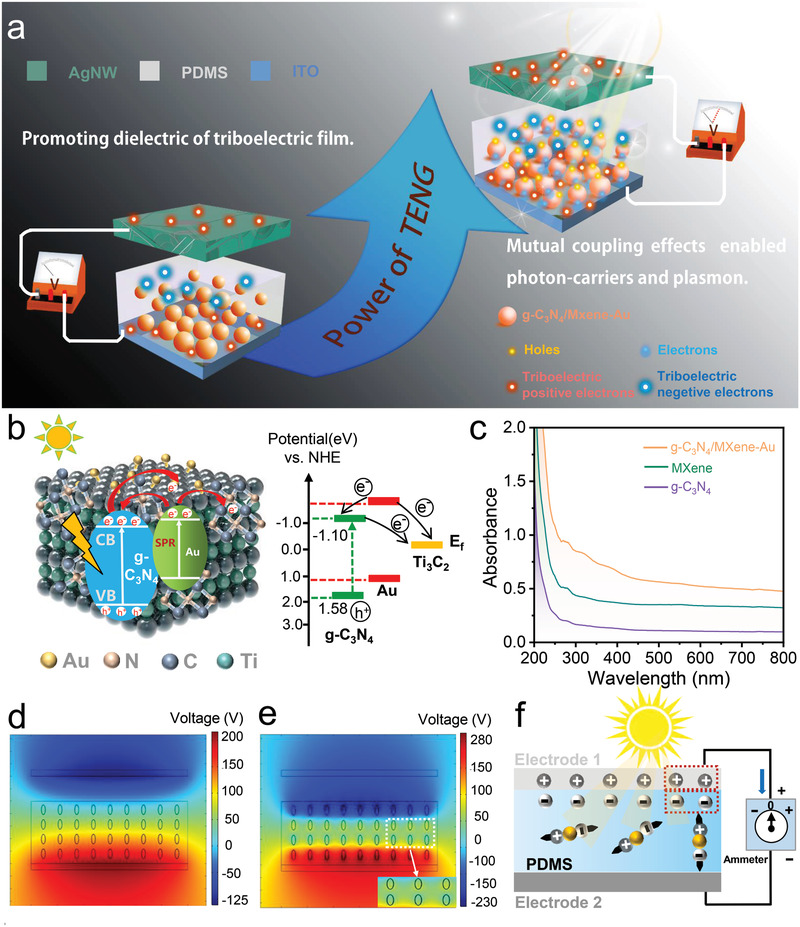
The theoretical model of the boosting output performance of TENG via charge traps from 2D materials g‐C_3_N_4_/MXene‐Au composites. a) The method of boosting output performance of the TENG in this research. b) The mechanism for setting more charge traps. c) The UV–vis spectrum of the g‐C_3_N_4_, Mxene, and g‐C_3_N_4_/MXene‐Au composites. d) The theoretical model of the charge traps boosted‐TENGs. e,f) The simulation of the outputs of the TENGs with composites is contrasted before and after lighting.

Here, we proposed a unique strategy to boost the performance of the TENGs via charge traps from photon‐generated carriers coupling surface plasmon effect enabled the way of g‐C_3_N_4_/MXene‐Au carbon cluster composites, which is sensitive to the wideband of visible light, as diagramed in the **Figure** **1**a. The semiconductor material g‐C_3_N_4_ absorbs the light energy then generates the carrier as the charge traps, and the surface plasmon resonance of the precious‐metal Au and the superior electronic transmission capability of MXene promoting more charge traps formation. In addition, the hot electron–holes excited by Au also form the charge traps. Then the charge traps via mutual coupling effects boost the surface charges accumulation of TENG, and the boosting TENG achieves a output voltage of 510 V, a current of 80 µA, and a highest peak power of 20 mW (4 cm × 4 cm) at the impedance of 2 MΩ. The output power density is 12.5 µW mm^−2^. Additionally, the impedance of the boosting TENG has a 50% reduction when compared to the normal TENG with PDMS. Furthermore, the sterilization system based on TENG is fabricated by utilizing self‐cleaning properties of g‐C_3_N_4_/MXene‐Au composites, and the disposal system is proven to be effective through the experiments. This research explores a new method to enhance the performance of the TENG via charge traps from mutual coupling effects and provides a feasible research direction for the high output electricity TENG fabrication.

## 2 Results and Discussion

The semiconductor has the properties of photon‐generated carriers at the optical irradiation, but the hole‐pairs from carriers are always in the recombination process, which induces the great reduction of charge traps and the enhancement ability to weaken for TENGs.

The quantum conversion efficiency in semiconductors can be expressed as

(1) $$I_{\mathrm{QE}}=\frac{1240\times I_{\mathrm{SC}}}{\lambda \times P_{\mathrm{abs}}}$$

where *I*_sc_ is the photocurrent density, *λ* is the wavelength of the incident light, and *P*_abs_ is the light intensity absorbed by the triboelectric film. Thus, the *I*_QE_ can be optimized by enhancing the photocurrent density, and the magnitude of *I*_sc_ is strongly linked with the properties of the carrier transport in the regions of triboelectric films. The higher the separation efficiency of the carriers is, the larger *I*_sc_ will get, and the more charge traps will generate in the region of tribo‐materials that the high output performance TENG can be obtained.

The graphite phase carbon nitride (g‐C_3_N_4_), possessing the advantages of stability, the wide bandgap, and sensitivity to visible light, has been receiving increasing attention from researchers. But the photogenerated electron–hole pairs produced by g‐C_3_N_4_ have a high recombination rate and small specific surface area, which seriously affects the practical application of g‐C_3_N_4_. The noble metal nanoparticles are generally chosen to combine with the semiconductor and form a Schottky barrier at the point of interface contact. The Schottky barrier can effectively capture electrons and transfer them to the metal surface, and retain a large number of holes in the semiconductor, thus inhibiting photogeneration carrier recombination. So in other words, the g‐C_3_N_4_ absorbs high‐energy photons, then excited into electrons and holes with visible light. The electrons are excited and move to the conduction band of g‐C_3_N_4_, while the holes remain in the valence band of that (Equation (2)). The generated electrons from the g‐C_3_N_4_ conduction band will flow into the electric field of MXene, and forming an upward‐bending band (Equation (3)). Reasons can be accounted by the fact that the MXene exposed to –O/–OH on the surface has a lower work function and the Schottky barrier forms at the contact interface of MXene and g‐C_3_N_4,_ as proved in Figure S1 (Supporting Information), the *I*/*V* curves of four materials are investigated. The above process advances the disjunction of photogenerated electrons and holes. In addition, the precious metal Au enabled the surface plasma effect to generate hot electron–holes and extend the visible light absorption of the g‐C_3_N_4_/MXene‐Au composites. The generated hot electrons will quickly occupy the empty conduction band above the Ag Fermi level (Equation (4)) and generate a new one. Since the new Fermi level of Au is higher than the redex potential (1.50 eV) of the g‐C_3_N_4_ conduction band and the Fermi level of MXene, hot electrons will be transferred from Au nanoparticles to g‐C_3_N_4_ and MXene, which are in close contact with Au (Equation (5)). Also, the photocurrent intensity was tested and depicted in Figure S2a (Supporting Information), the MXene has a low photocurrent as irradiated from visible light, secondly is g‐C_3_N_4_ and next is the g‐C_3_N_4_/MXene composites, the Au/g‐C_3_N_4_/MXene composites. It turns out that composition effect has a better photoelectric conversion efficiency, and moreover, the Raman of the composite is also tested, the result is shown in Figure S2b in the Supporting Information. Thus, the electrons transfer achieving, and the separation of photo‐electrons from composites can be effectively promoted, and more charges traps will be obtained to boost the output performance of the TENGs, as Figure 1b depicted.

(2) $$g-C_{3}N_{4}+hv\to g-C_{3}N_{4}\left(e^{-}+h^{+}\right)$$

(3) $$g-C_{3}N_{4}\left(e^{-}\right)+\mathrm{MXene}\to g-C_{3}N_{4}+\mathrm{MXene}\left(e^{-}\right)$$

(4) $$\mathrm{Au}+hv\to \mathrm{Au}(e^{-})$$

(5) $$\begin{array}{ccc} & & \mathrm{Au}\left(e^{-}\right)+g-C_{3}N_{4}+\mathrm{MXene}\to \mathrm{Au}+g\\ & & \;-C_{3}N_{4}\left(e^{-}\right)+\mathrm{MXene}\left(e^{-}\right) \end{array}$$

The UV–vis diffuse reflection is adopted to analyze the optical properties of the samples to verify the theory set forth above. As shown in Figure 1c, the absorption edge of g‐C_3_N_4_ is about 470 nm, and the absorption edge redshift is observed of the g‐C_3_N_4_/MXene‐Au composites. This is due to the heterojunction structure formed by the interface of Au, g‐C_3_N_4_, and MXene, which changes the Fermi level of g‐C_3_N_4_. The energy of the bonding electron in the g‐C_3_N_4_ conjugated system decreases when it transitions from the ground state to the antibonding π* orbital. Thus, the absorption peak redshift is realized, and the light absorption properties of the composite are greatly improved on the condition of visible light range, which is the result of the excellent light absorption ability of MXene. In addition, one further point demonstrates that the plasmon resonance of Au plays a role. The bandgap of the semiconductor is obtained from the UV–vis DRS (Figure 1c) spectrum through the tangent method according to the Equation (6).

(6) $$E_{g}=1240/\lambda_{g}$$

The unit of *λ*_g_ is nm, while the unit of *E*_g_ is eV. It is believed that the band edge wavelength (*λ*_g_) of the semiconductor is determined by the bandgap width (*E*_g_). The energy gap of the g‐C_3_N_4_ /MXene‐Au sample is about 2.2 eV, which is significantly smaller than that of g‐C_3_N_4_ at 2.7 eV. That also means the more charge traps will construct in the region of tribo‐materials, and the higher output electric of TENG will achieve.

The theoretical model of boosting the output performance of TENGs via charge traps from mutual coupling effects is constructed, and the process is displayed in Figure 1d and Figure S1 (Supporting Information). The vertical contact–separation mode of TENG was utilized to introduce the enhancing mechanism. The first layer is the Ag nanowire electrodes attached to PMMA as one triboelectric layer, and the other layer is PDMS mixed with g‐C_3_N_4_/MXene‐Au composites spunning on the ITO glass. The electrons–holes via mutual coupling effects can be excited within the triboelectric film and distributing at the interface of PDMS and composite materials (Figure S3, Supporting Information). The mutual contaction occurred at the interface of electrode one and dielectric film and generated the same amount of charges with opposites signs with the external force. At the time of external force withdraw, electrode 1 separated from the dielectric film, and the electrons would flow from electrode 1 to electrode 2. The continuance forward current of the TENG is generated, and the first layer is moved to the original position. An electric field is set up as a result of opposite charges accumulate on the surface of electrode one and dielectric film. Thus, the electron–holes generated from mutual coupling effects can be vertically arranged, and the charge traps formed to enhance the ability to capture electrons during contacting‐separating progress. While the force is applied again, a reverse current is generated as a result of electrons flowing from top electrode to bottom electrode. When electrode 1 retouched the dielectric film, the electric field would disappear. However, according to the effect of dielectric hysteresis, the vertical photon‐generated pairs would not completely dissipate. That means more net converse charges would be accumulated on electrode one and the dielectric film, and a periodic current will be generated in the process of periodical contact separation. In addition, the output voltages of TENG‐filled g‐C_3_N_4_/MXene‐Au composites with visible light were simulated through COMSOL 5.3a to verify the method we have put forward. And also, the voltage without visible light has been simulated as a contrast. The results indicated that the output circuit of TENG with composite materials but without visible light was six times higher than the blanker controller, and the voltage of TENG with composites together with visible light was two times larger than the TENG only with composites. The results are depicted in the Figure 1d,e, these results provide theoretical verification for our subsequent experiments. Also, the output impedance decreased with the charge traps.

**Figure 2:**
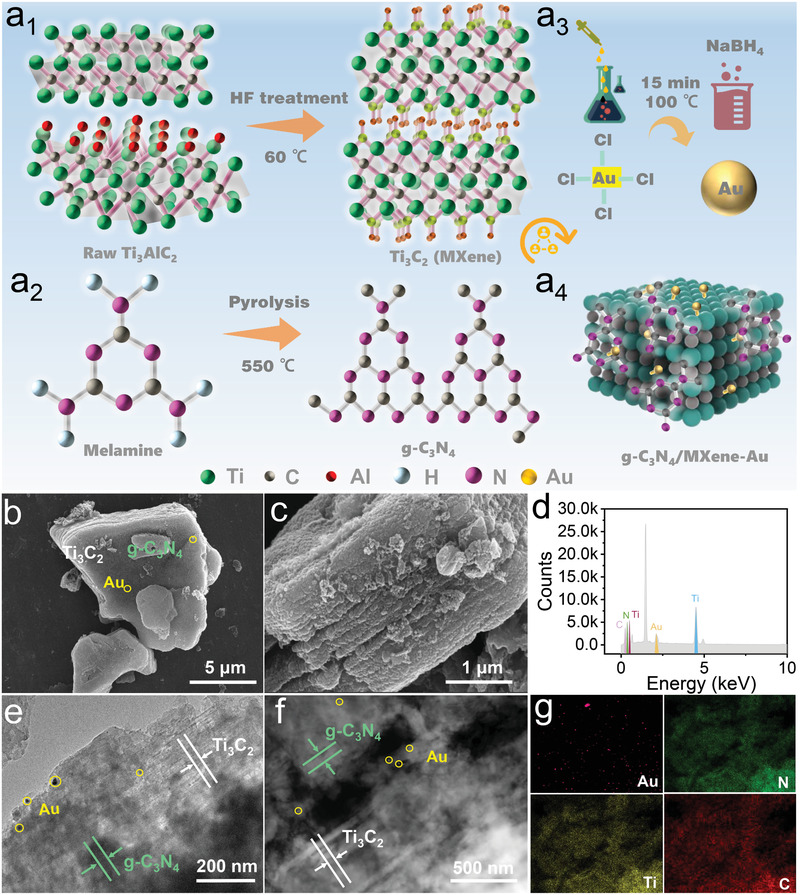
The preparation and characterization of the g‐C_3_N_4_/MXene‐Au composites. a_1_–a_4_) The preparation process of the g‐C_3_N_4_/MXene‐Au composites. b,c) The SEM images of the composite. d) The result of the XRD of the composite. e–g) The TEM image and element characterization of the composite.

MXene was prepared by chemical wet etching, and the process is diagrammed in **Figure** **2**a. Ti_3_AlC_2_ (10.0 g) powder with a purity of 98% was immersed in 100 mL of HF solution with a concentration of 49%. The Ti_3_AlC_2_ powder was added in small amounts and multiple times, then the etching reaction placing a magnetic stirrer in the beaker, and stirring continued under ambient temperatures for 24 h. The reaction equation of the Ti_3_AlC_2_ etching process as follows^[^ ^29^, ^30^, ^31^, ^32^ ^]^

(7) $${\mathrm{Ti}}_{3}{\mathrm{AlC}}_{2}+3\mathrm{HF}={\mathrm{AlF}}_{3}+{3/2H}_{2}+{\mathrm{Ti}}_{3}C_{2}$$

(8) $${\mathrm{Ti}}_{3}C_{2}+{2H}_{2}O={\mathrm{Ti}}_{3}C_{2}{\left(\mathrm{OH}\right)}_{2}+H_{2}$$

(9) $$\mathrm{MXene}+2\mathrm{HF}={\mathrm{Ti}}_{3}C_{2}F_{2}+H_{2}$$

The samples were naturally cooled to room temperature after the reaction that was centrifugally cleaned with deionized water until pH was nearly neutral. Then cleaned with ethanol three times and dried overnight in a vacuum drying oven at 60 ℃, and the final black powder was MXene. The page of scanning electron microscope (SEM) is illustrated in Figure S4 in the Supporting Information.

The preparation of g‐C_3_N_4_ material was through the method of classical high‐temperature calcination, and material synthesis and processing technology was depicted as follows shown in Figure 2a_2_: Take 10.0 g of melamine raw material and place it in a tube furnace, set the program to heat to 550 °C at a heating rate of 3 °C min^−1^, and keep it at this temperature for two hours. After the reaction is completed, it is naturally cooled to room temperature and finally ground into a pale‐yellow g‐C_3_N_4_ powder.^[^ ^33^, ^34^, ^35^ ^]^ The SEM page is shown (Figure S5, Supporting Information).

The g‐C_3_N_4_/MXene is prepared via the ultrasonic‐assisted high‐temperature calcination method: select 0.2 g MXene and 0.8 g g‐C_3_N_4_ to ultrasonically mix. After the two substances were uniformly mixed, continue to stir the solution on a magnetic stirrer until the ethanol was completely volatilized and get the dark gray powder. Then put the powder into a tube furnace, heat it to 150 ℃ at a heating rate of 3 ℃ min^−1^ in N_2_ atmosphere, and keep it at this temperature for 30 min to obtain the g‐C_3_N_4_/MXene composites.^[^ ^36^, ^37^ ^]^

The nanosized gold spheres were prepared by citrate reduction and mixed with the g‐C_3_N_4_/MXene in the deionized water. Then a uniform suspension was formed via ultrasounding for 30 min, passed N_2_ into the suspension continuously, and stir in the dark for one hour. After the reaction, the mixture was centrifuged and cleaned several times and then dried in a vacuum at 60 °C. ^[^ ^38^ ^]^ The sample obtained was g‐C_3_N_4_/MXene‐Au, as shown in Figure 2a_4_. In addition, the phase, composition, and structure of the composite were analyzed through the characterization of SEM, Transmission Electron Microscope (TEM), and diffraction of x‐rays (XRD), which verified that the material was prepared successfully through the method mentioned above. The size and shape of the composite are diagramed in Figure 2b–g.

**Figure 3:**
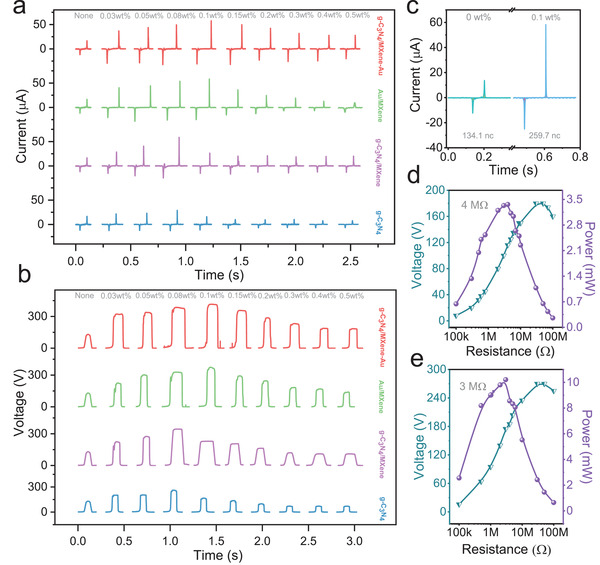
The output performance of the TENG with g‐C_3_N_4_/MXene‐Au composites in normal condition. a) The short‐circuit current of the TENG with composites. b) The open‐circuit voltage of the TENG with composites. c) The charges of the TENG with composites contrast with the normal TENG with PDMS. d) The output power of the normal TENG with PDMS. e) The output power of the TENG with composites.

To verify the mutual physics effect is superior to the single effect for enhancing the output electricity of TENG. The materials of g‐C_3_N_4_, g‐C_3_N_4_/MXene, MXene/Au, and g‐C_3_N_4_/MXene‐Au were chosen to verify the mechanism of more charge traps possess the better capability to boost output electricity TENG. While the ups and downs of output electric for TENGs are filled with composites, which changes the dielectric properties of the triboelectric film. And the variation of output electric for TENGs with the materials without light radiation are investigated in **Figure** **3**a,b. The doping content was chosen from 0 to 0.5 wt% by validating a lot of the experimental data, and four different materials were doped into PDMS abiding by the same experimental procedures. And the procedure to make the composite triboelectric film is shown in Figure S6 in the Supporting Information. Also, the XRD was tested in Figure S7 (Supporting Information) to verify the adhesions of composites with PDMS. Then the current of mixed TENGs was measured through electrometer (Keithley 6514), and the current all increased firstly and then decreased. Reason is that filling nanoparticles have changed the dielectric properties of triboelectric film firstly, and then the percolation phenomenon will appear as the mixed content increases. While the size and conductivity of mixed materials are different, the point that reaches the highest output electricity are not at the same point. As Figure 3a depicted, the output current generated from tribo‐material mixed with g‐C_3_N_4_ achieved the minimum output current as a result of the lowest electrical conductivity compared to other nanometer materials, and the highest output current is about 35 µA at the doping content of 0.08 wt%, which is 2.5 times larger than the output current of the triboelectric film only with PDMS (13 µA). The output current of TENG with g‐C_3_N_4_/MXene is higher than that only with g‐C_3_N_4_. The optimal output current is about 50 µA with a doping content of 0.08 wt%. This comes from the excellent electronic transmission ability and electrical conductivity of MXene. Thus, the TENG constructed with triboelectric film filling in g‐C_3_N_4_/MXene/Au obtain the highest current, which is about 60 µA at a mixing content of 0.1 wt%. And the TENG with MXene/Au is about 56 µA, close enough to the output current of TENG with MXene/Au, but the output drops sharply over the proper doping point. In addition, the current of TENG with g‐C_3_N_4_/MXene/Au composites is more stable than that with MXene/Au over the optical doping density. Moreover, the output voltage has the same numerical trend as the output current. The highest output voltage of TENG is about 413 V which is obtained with doping material of g‐C_3_N_4_/MXene‐Au at the content of 0.1 wt%, and the output voltage of TENG with MXene/Au is 374 V. the TENG with g‐C_3_N_4_/MXene is 343 V, and the voltage just with g‐C_3_N_4_ is 256 V, and the optimum voltage of TENG with these two materials both reached at the doping density of 0.08 wt%, as shown in the Figure 3b.

Also, the charges of TENGs of doping 0 and 0.1 wt% are calculated to verify the doping particles have an effect on boosting the output performance of TENG. The results are shown in Figure 3c; the generated charge of TENG with 0.1 wt% doping is about 259 nC, which is about two times larger than the TENG without doping. The peak output power of TENG without doping and the TENG with g‐C_3_N_4_/MXene‐Au at optimum doping density were measured. The peak output power of TENG without doping away from 0.7 mW up to 3.4 mW as the impedance ranged from 100 kΩ to 4 MΩ and then decreased as the impedance increased, which indicates that the peak value located at the resistance of 4 MΩ, as shown in the Figure 3d, the peak output power of TENG without doping as a contrast. While the power of TENG with doping at the optimum content reached the maximum value of 10 mW as the resistances range from 100 kΩ to 3 MΩ, the power goes down with the increase of resistances and the results were diagramed in Figure 3e. The above results merely utilized the electrical conductivity of doping‐particles to boost the output of TENG, and the optical properties of the composites to enhance the output electric of TENG will be investigated in the next session.

**Figure 4:**
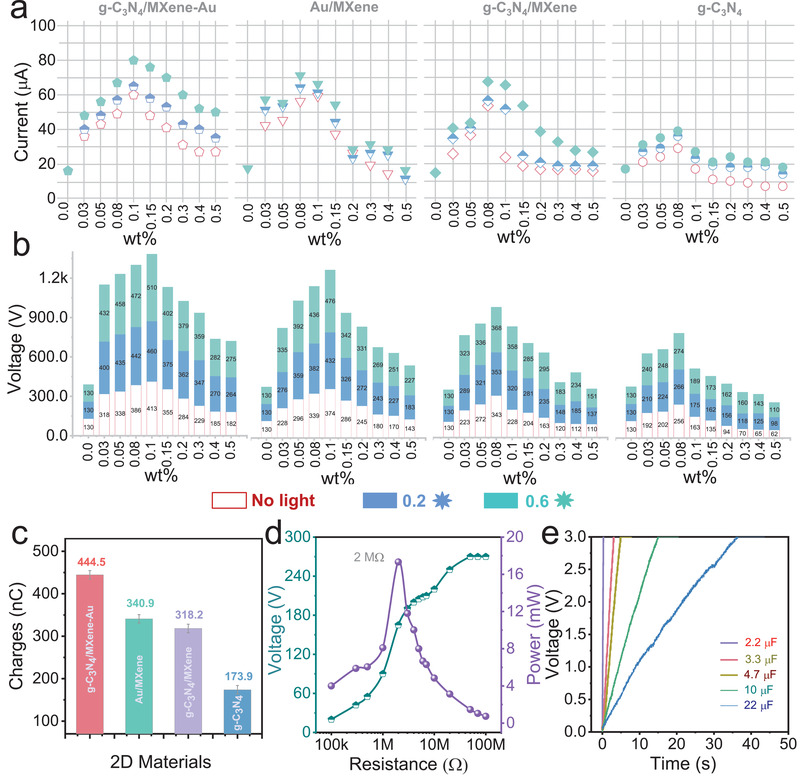
The output performance of the TENGs with composites under visible light irradiation. a) The short‐circuit current of the TENGs with the materials of g‐C_3_N_4_, g‐C_3_N_4_/MXene, MXene/Au, and g‐C_3_N_4_/MXene‐Au under the 0.6 times visible light irradiation. b) The open‐circuit voltage of the TENGs with g‐C_3_N_4_, g‐C_3_N_4_/MXene, MXene/Au, and g‐C_3_N_4_/MXene‐Au. c) The charges of the TENG four materials with the visible light. d) The output power of the TENG with g‐C_3_N_4_/MXene‐Au composites under visible light. e) The charge curve of the TENG with g‐C_3_N_4_/MXene‐Au composites under visible light.

The mutual coupling effects have a promotion impact on boosting the output electricity of TENG in contrast with a single effect; the light radiation was applied on the working TENG until the output electricity of TENGs reaches saturatiton at the normal condition. A high‐intensity discharge lamp was employed as the excitation for photon‐generated carriers and surface plasmon from Au nanoparticles. The lamp was calibrated by a silicon reference cell to obtain a light intensity of 60 mW cm^−2^ as 0.6 times the sunlight. Also, a 400 nm cut filter was added to obtain the visible light excitation to enhance TENG. Experiments of two different intensity light (0.2 times, 0.6 times of sunlight) were carried out to investigate the influence of light‐intensity variation on output electricity. The results were diagramed in **Figure** **4**a,b, the output electricity of TENGs with four materials all magnified with the increase of light intensity. While the boosting output via photon‐generated carriers from g‐C_3_N_4_ as the charge traps have the weakest enhancement effect, the output voltage of 274 V was obtained that only an improvement of 20 V was got over the unilluminated device. In addition, the current of this TENG is only about 40 µA, the photon‐generated carriers from g‐C_3_N_4_ are partial to recombine, and the charge traps decrease which is the main reason for limiting the boosting ability to the TENG. Thus, the MXene was chosen to improve the electrons‐holes separation rate of g‐C_3_N_4_ with the visible light and obtained more charge traps for boosting the output electricity of TENG. The results of 40 µA (short‐circuit current) and 368 V (open‐circuit voltage), two times larger than the output electricity of TENG with g‐C_3_N_4_, verified the effectiveness of the method. Moreover, another effect of surface plasmon enabled MXene/Au was utilized to heighten the output performance, and a high current of 70 µA and a voltage of 476 V were achieved via the TENG with Au/MXene. While the output decreased sharply as a result of permeation composites in two‐phase fluid, and the charge leakage occurred at the surface of the triboelectric film. From this, the g‐C_3_N_4_/MXene‐Au composites filled into blank triboelectric film with visible light possess the optimal output performance, a sustainable output of 510 V, 80 µA were generated via TENG with composites. And the output was stable and high even over the optimum doping content, which verified that g‐C_3_N_4_/MXene‐Au composites with visible light could generate more charges traps, and a higher output electricity of TENG will achieve. The boosting was recorded in Video S1 in the Supporting Information.

Furthermore, the charges were calculated from the integral of current over one period, and the results were diagramed in the Figure 4c. The TENG with g‐C_3_N_4_/MXene‐Au composites at the radiation of visible light got the top charge value (444.5 nC) and output of TENG only with g‐C_3_N_4_ was minimum, the variation consistent with the trend of current and voltage. The output power of TENG based on g‐C_3_N_4_/MXene‐Au composites with light radiation was also measured with the external impedance range from 100 kΩ to 100 MΩ, as illustrated in Figure 4d. The peak power of TENG is nearly 20 mW at the impedance of 2 MΩ, the definite growth on peak power and a reduction of the impedance was achieved via multi‐physical effects. Indeed, the TENG based on g‐C_3_N_4_/MXene‐Au composites with light radiation is over sixfold increase in output power and a 50% reduction in the impedance in contrast with a TENG only with PDMS triboelectric film, and the reason for this is illustrated in Figure S8 in the Supporting Information. Subsequently, the charging rate was studied to verify the excellent output of the TENG, and the results were presented in Figure 4e. And also, the boosting TENG had excellent electrical output, the 100 LEDs in parallel were lighted and the self‐powered temperature sensor node was realized in Figure S9 and Video S2 (Supporting Information), respectively.

**Figure 5:**
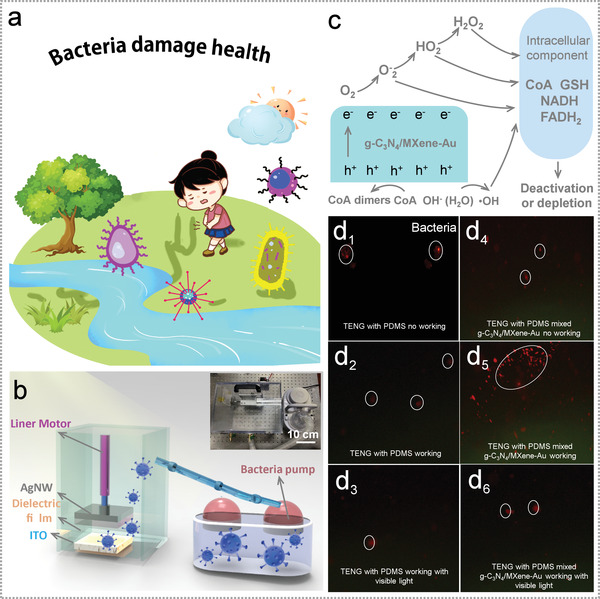
The sterilization system constructs based on TENG. a) The damage of the bacteria to human health. b) The system chart and image of the sterilization system. c) The mechanism of the sterilization process. d_1_–d_6_) The contrast of the sterilizing effect of TENG with composites and PDMS.

Salmonellosis is the main pathogen leading to food poisoning and widely exists in the environment, especially dairy products, meat, eggs, and fresh fruits and vegetables are highly susceptible to contamination. Salmonella infection is not usually life‐threatening, but for certain people, especially infants, young children, and people with weakened immune systems, the development of complications can be dangerous,^[^ ^39^, ^40^, ^41^, ^42^, ^43^ ^]^ the **Figure** **5**a diagrams the threatens and the morphology of the bacteria. Thus, developing a creative and simple method to kill bacteria is hot research concerning human health. Here, a sterilization system via TENGs was constructed in Figure 5b. The system is made up of TENG (with 1 wt% composites which can be exposed to the air atmosphere), a few salmonellosis, and a bacteria bump, also a liner motor providing the continuous and steady applied force. The salmonellosis was pumped to space where the TENG set, and we adopt the simple variable method to verify that the TENG with high doping concentration g‐C_3_N_4_/MXene‐Au composites was effective in sterilization. The photogenerated hole‐electrons exposure to the air environment, and photogenerated holes and reactive oxygen species (·OH, O_2_^−^, HO_2_, H_2_O_2_), reacts directly with the components of the cell and achieve the purpose of bacteria inactivation. In addition, the number of bacteria was detected by fluorescence method. The salmonellosis was adopted to verify the sterilization system, and the microphotograph of the salmonella enterica is shown in the Figure S10 in the Supporting Information. As shown in Figure 5d_1_–d_6_, the TENG with composite material and only adopt PDMS as dielectric material were compared. Figure 5d_1_–d_3_ diagrams the bacterial on the TENG only with PDMS at different work conditions, while Figure 5d_4_–d_6_ shows the TENG mixed with composites. The TENG with PDMS showed small electricity in a state of inactivity, and fewer bacteria landed on the surface of PDMS, as presented in Figure 5d_1_. And more bacteria were adsorbed onto the surface of PDMS by the interaction of static electricity when the TENG started to work, but the bacteria were presented in smaller quantities as a result of the low output of the TENG with PDMS. While the number of bacteria had decreased with the visible light radiation due to the fact that has a certain bactericidal effect. By contrast, the TENG with composites possessed the high output electricity would attach more bacteria when contact–separation mode started. And the number of bacteria had dried up mainly by reason of oxidizability derived from g‐C_3_N_4_/MXene‐Au composites. The presentation of this antibacterial test is shown in Video S3 in the Supporting Information. Thus, a new, portable and effective way to sterilize salmonellosis was demonstrated successfully via the TENG.

## 3 Conclusions

In conclusion, a new strategy of charge traps generated from photon‐generated carriers and surface plasmon mutual coupling effects enabled g‐C_3_N_4_/MXene‐Au composites is proposed to boost the output performance. The composites are prepared via the methods of etching and drying, also g‐C_3_N_4_, g‐C_3_N_4_/MXene, and MXene/Au doping materials enhancing performance based on only one physical effect are prepared as contrasts. The charge traps from mutual coupling effects for boosting performance are analyzed theoretically and verified by experiments. The results show that the output performance of the TENG boosting via mutual effect is superior to the single effect. The highest open‐circuit voltage of 510 V, short‐circuit current of 80 µA, and output power of 20 mW were achieved, which was six times larger than that of a conventional TENG with PDMS. The output power density is 12.5 µW mm^−2^. Moreover, a sterilization system was demonstrated based on TENGs utilized the self‐cleaning feature of g‐C_3_N_4_/MXene‐Au composites. This work provides insights and a promising avenue for boosting output electricity of the vertical contact‐separation mode TENG.

## 4 Experimental Section

### Fabrication of the Triboelectric Film via Doping PDMS with Four Materials

The preparation process of triboelectric films doping four materials mainly adopted the four instruments including deaeration mixer (THINKY MIXER AR‐100), glue homogenizer (EasyCoater 4, Schwan), and vacuum drying oven, and the detailed flow is shown in Figure S5a in the Supporting Information. At great length, the PDMS combination was got from Sylgard 184, were deeply mixed in the proportion of 10:1, and then the mixture was stirred by a mixmaster for 5 min to obtain a homogeneous mixture. Four materials of g‐C_3_N_4_, g‐C_3_N_4_/MXene, MXene/Au, and g‐C_3_N_4_/MXene‐Au doping into PDMS with nine contents, and then repeat the previous stirring step. Then the agitator was set as the mode for 2 min to remove the bubbles in the mixture. Then the mixture was attached onto the ITO glass (4 cm × 4 cm) via the method of spin coating at 350 rpm for 120 s. Finally, the composite was adopted the oven drying method to film.

### Fabrication of the Lighting System

The lamp that provides light radiation (CEL‐HXF300‐T3) and the lamp was put onto the lifting platform (LFP200) set as a 60° radiation source. Also, a filter (UVIRCUT400) was added to the lamp to generate visible light for boosting the output electricity of the TENGs.

### Characterization

The outputs electricity of the TENGs were obtained by an electrostatic meter (Keithley model 6514) and a data acquisition card (NI PCI‐6259) which was fixed on a computer, and the external sustained motivation was supplied by a linear motor (DIY‐37GB330).

### Statistics

The output performance (output current and output voltage) were all adopt the original data, and the data were chosen as the average of 50 sets of data. The charges were calculated through the current in one cycle via the software of Matlab 2015a. And the output powers were calculated through the formula *P = U* ^2^_load_/*R*, all of the data curves were diagramed by the software of Origin Pro(Learning).

## Conflict of Interest

The authors declare no conflict of interest.

## Supporting information

Supporting Information

Supplemental Video 1

Supplemental Video 2

Supplemental Video 3

## Data Availability

Research data are not shared.
