# Supplementary material for: Boosting Output Performance of Triboelectric Nanogenerator via Mutual Coupling Effects Enabled Photon‐Carriers and Plasmon
Source: Adv Sci (Weinh). 2021 Nov 23;9(4):2103957. doi: 10.1002/advs.202103957 (PMC8811832; doi:10.1002/advs.202103957)
Supplement: Supplementary file 1 — Supporting Information [file ADVS-9-2103957-s002.pdf]

ADVANCED SCIENCE
Open Access

Supporting Information

for *Adv. Sci.,* DOI: 10.1002/advs.202103957

Boosting output performance of triboelectric nanogenerator via mutual coupling effects enabled photon-carriers and plasmon

Xin Chen, Yanjun Zhao, Fayang Wang, Daqiao Tong, Lingxiao Gao, Dongxiao Li, Liangke Wu, Xiaojing Mu* and Ya Yang**

Supporting Information for

Boosting output performance of triboelectric nanogenerator via mutual coupling effects enabled photon-carriers and plasmon

Xin Chen, Yanjun Zhao, Fayang Wang, Daqiao Tong, Lingxiao Gao, Dongxiao Li, Liangke Wu, Xiaojing Mu* and Ya Yang**

[*] X. Chen, F. Y. Wang, D. X. Li, Y.J. Zhao, D.Q. Tong, Prof. X. J. Mu, Key Laboratory of Optoelectronic Technology & Systems Ministry of Education, International R & D center of Micro-nano Systems and New Materials Technology, Chongqing University, Chongqing, 400044, China
E-mail: mxjacj@cqu.edu.cn
X. Chen, Prof. Y. Yang, CAS Center for Excellence in Nanoscience, Beijing Key Laboratory of Micro-nano Energy and Sensor, Beijing Institute of Nanoenergy and Nanosystems, Chinese Academy of Sciences, Beijing, 101400, China
E-mail: yayang@binn.cas.cn
L. X. Gao, School of Mechanical Engineering, Hebei University of Technology, Tianjin, 300401, P.R. China
E-mail: lingxiao.gao@hebut.edu.cn
L. K. Wu, College of Aerospace Engineering, Chongqing University, Chongqing, 400044, China

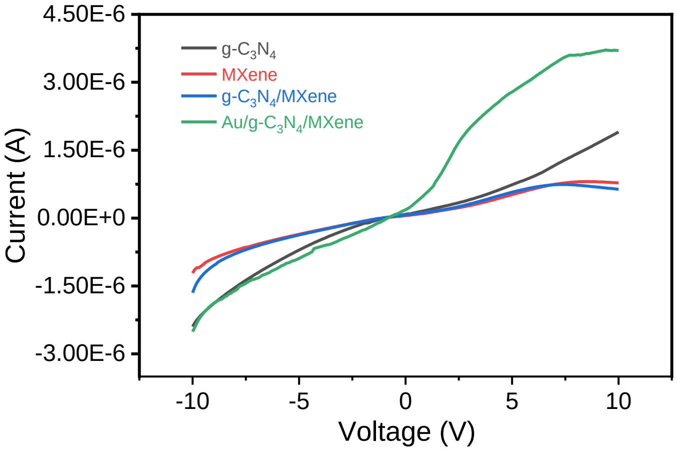

Figure S1. The I/V curve of the composite materials.

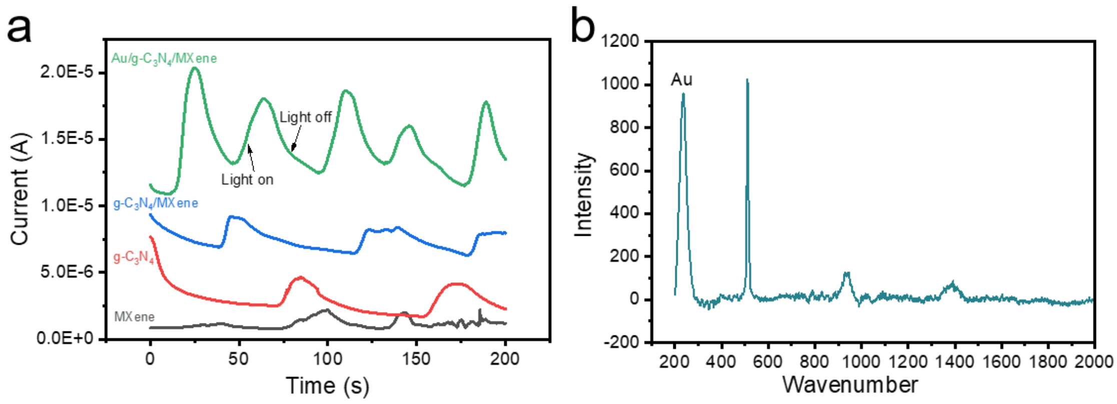

Figure S2. a) The photocurrent intensity of the composite materials. b) The Raman spectrum of Au/g-C_3N_4/MXene.

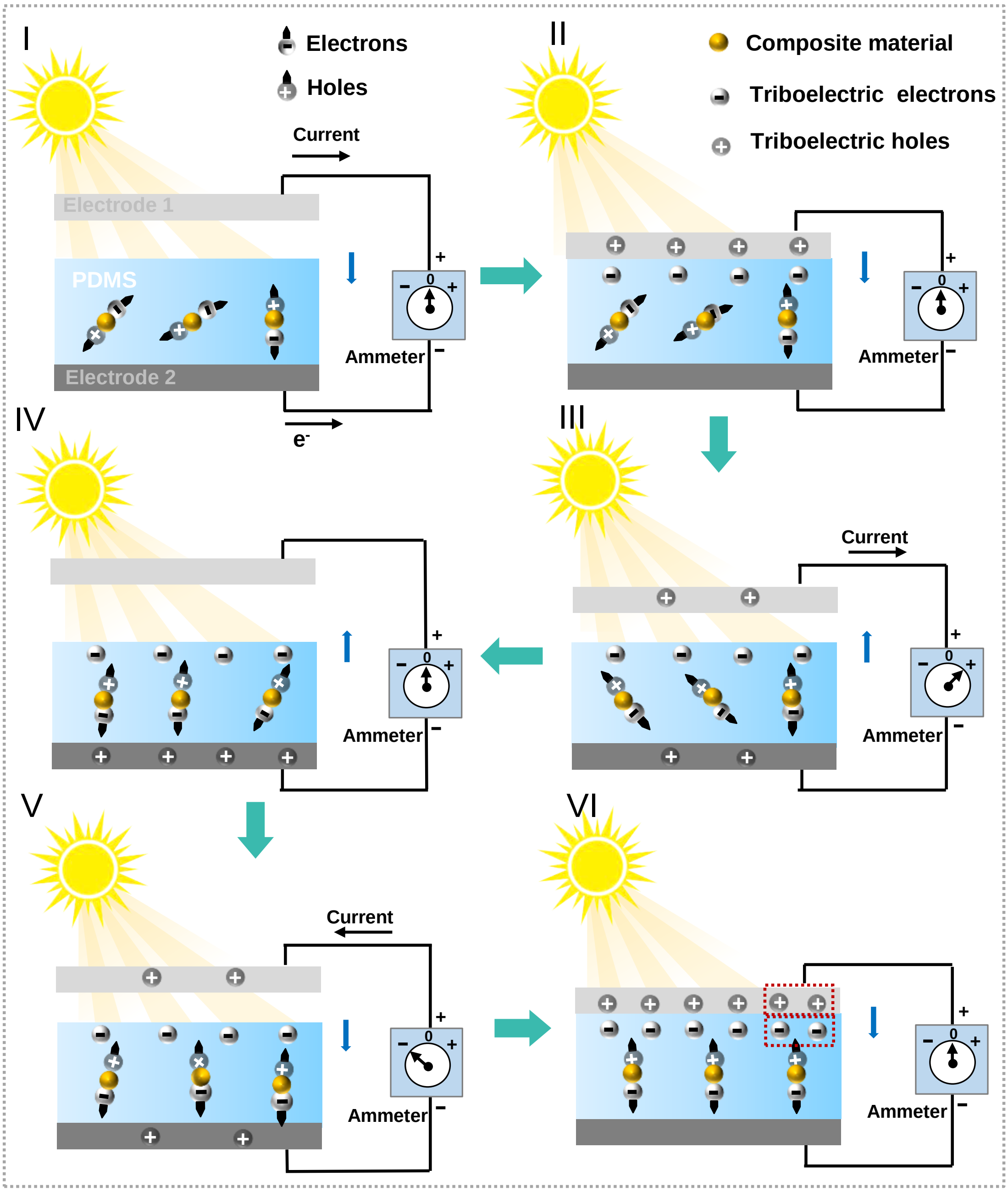

Figure S3. Theoretical model of the boosting output performance of TENG via charge traps from 2D materials g-C_3N_4/MXene-Au composites.

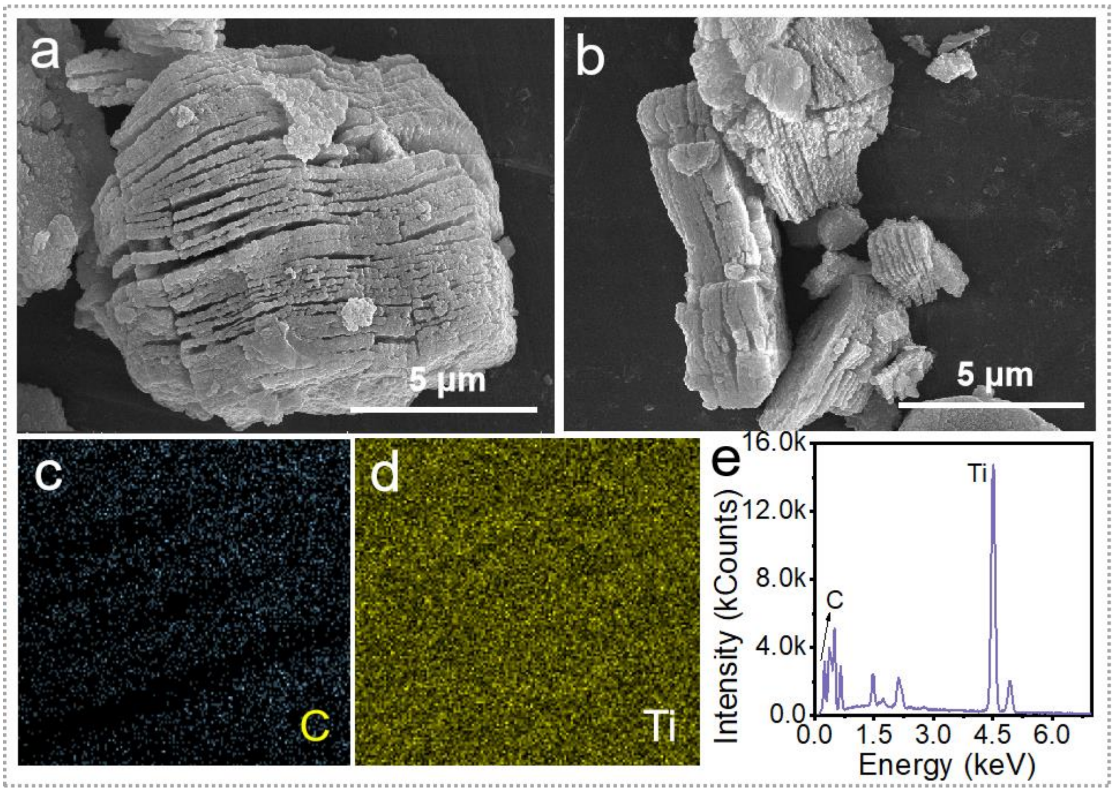

Figure S4. The SEM characteristics of the MXene. a-b) The SEM image of the MXene. c-e) The mapping results of the MXene.

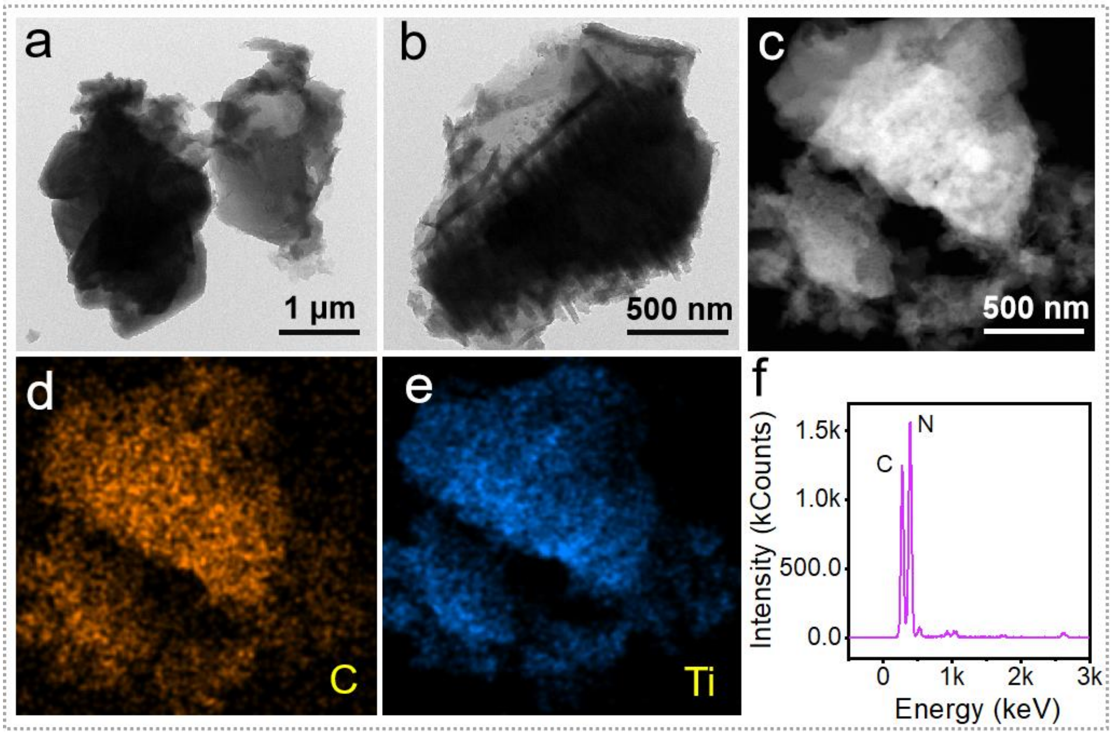

Figure S5. The TEM characteristics of the g-C_3N_4. a-c) The TEM image of the g-C_3N_4. d-f) The mapping results of the g-C_3N_4.

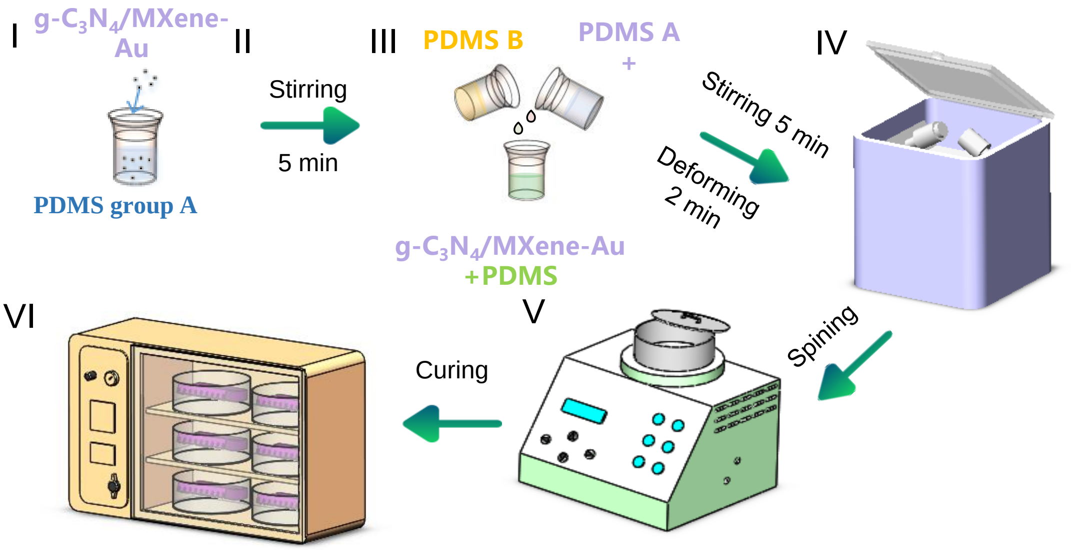

Figure S6. the procedure to make composite triboelectric film

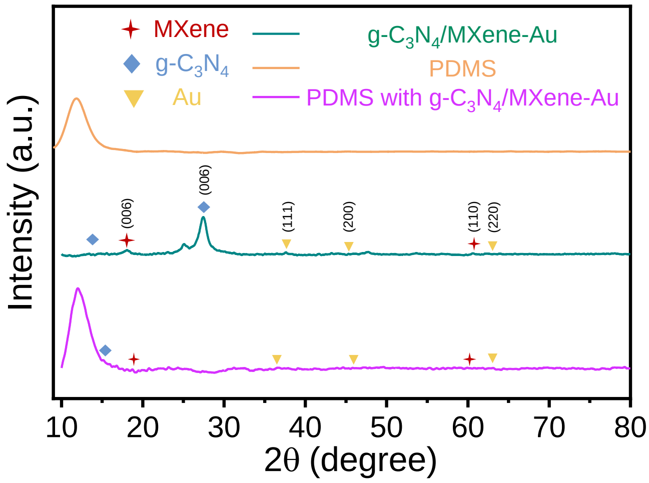

Figure S7. The XRD of the PDMS, the g-C_3N_4/MXene-Au composites and PDMS with g-C_3N_4/MXene-Au composites.

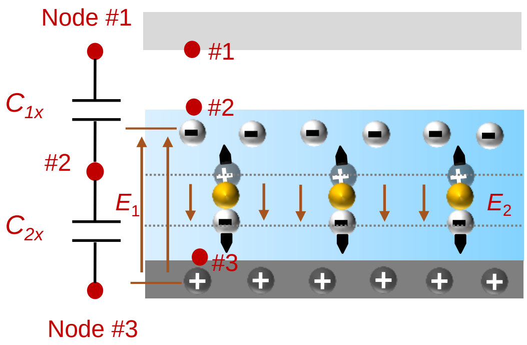

Figure S8. Theoretical model of vertical contact-isolation triboelectric nanogenerator.

Theoretical models for conductor-to-dielectric contact-mode TENG whose equivalent circuit is a series of two capacitors (C_1 and C_2) is depicted in Figure S6. When the equilibrium is reached, an electric field (E_2) will be established by the hot electron-hole pairs between dielectric film and metal foil 2, and the direction of this electric field will be opposite to that of generated by triboelectrification (E_1). The total field strength in the capacitor (C_2) is weakened, resulting in a reduced voltage (U) between dielectric film and metal foil 2. Assuming that the electric quantity (Q) of the capacitor (C_2) is constant, the capacitor (C_2) will increase, which can be obtained from formula (1):

$$C_2 = \frac{Q}{U} \tag{1}$$

The equivalent capacitance (C) of the TENG is

$$C = \frac{C_1 * C_2}{C_1 + C_2} \tag{2}$$

So an increase in C_2 will lead to an increase in C. The output impedance of the capacitor is

$$\mathbf{Z} = \frac{1}{j\omega C} \tag{3}$$

Hence, a reduction of the output impedance will be obtained.

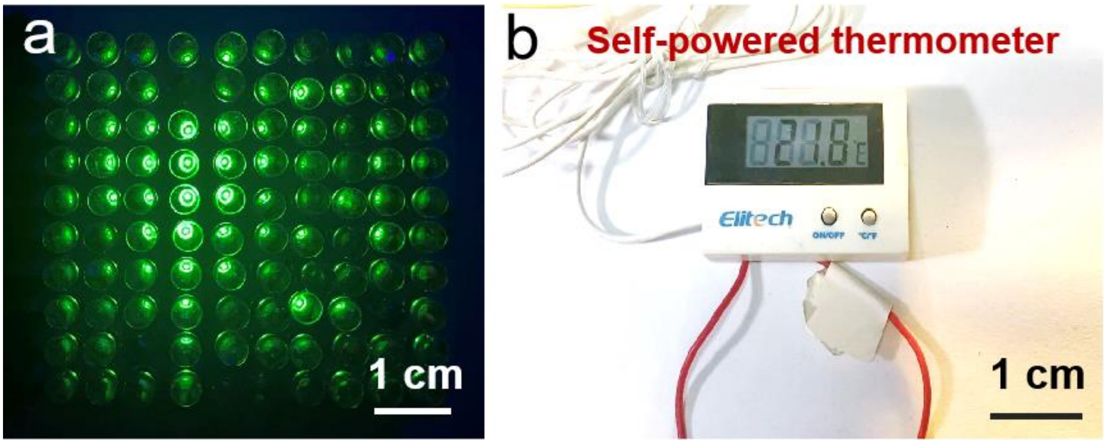

Figure S9. The self-powered thermometer was realized

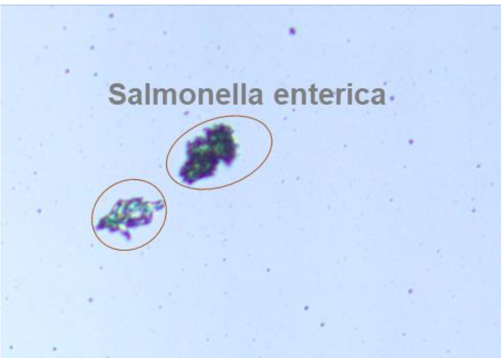

Figure S10. The microphotograph of the salmonella enterica.

Video S1: Boosting the output performance real-time via the charge traps from mutual coupling effect.

Video S2: The charge ability of the high performance of TENG.

Video S3: A sterilization system construct based on TENG.
